# Memory and attention during an alcohol hangover

**DOI:** 10.1002/hup.2701

**Published:** 2019-07-11

**Authors:** Lydia E. Devenney, Kieran B. Coyle, Joris C. Verster

**Affiliations:** ^1^ School of Psychology, Life and Health Sciences Ulster University Londonderry, Northern Ireland UK; ^2^ Division of Pharmacology Utrecht University Utrecht The Netherlands; ^3^ Institute for Risk Assessment Sciences (IRAS) Utrecht University Utrecht The Netherlands; ^4^ Centre for Human Psychopharmacology Swinburne University Melbourne Victoria Australia

**Keywords:** alcohol, attention, cognitive functioning, hangover, memory, mood

## Abstract

**Objective:**

This study aims to investigate attention, memory functioning, and mood in a natural setting with real‐life alcohol consumption levels.

**Methods:**

Seventy‐four participants with a mean (*SD*) age of 24.5 (7.0) years old participated in a naturalistic study. A between subjects design was applied comparing a hangover group with an (alcohol‐free) control group. Participants in the hangover group consumed a mean (*SD*) of 13.8 (10.2) alcoholic drinks the night before testing. Cognitive tests included the Stroop test, Eriksen's flanker test, a divided attention test, intradimensional–extradimensional set shifting test, spatial working memory test, and free word recall test.

**Results:**

The hangover group had increased reaction times compared with the control group. Selective attention (Stroop and Eriksen's Flanker test performance) was significantly impaired during alcohol hangover. However, the number of errors did not differ significantly between the groups in any task. Mood assessments revealed that the hangover group reported significantly higher levels of drowsiness and clumsiness compared with the control group.

**Conclusion:**

Selective attention was significantly impaired during alcohol hangover. The differences between the hangover and control group did not reach significance for other forms of attention or memory.

## 1 INTRODUCTION

An alcohol hangover is defined as the combination of mental and physical symptoms that are experienced the day after an episode of heavy alcohol consumption, starting when blood alcohol concentration (BAC) approaches zero (Van Schrojenstein Lantman, van de Loo, Mackus, & Verster, 2016). The alcohol hangover is characterized by various symptoms and complaints, including fatigue, nausea, and headache, that can occur simultaneously or sequentially (Penning, McKinney, & Verster, 2012; Verster et al., 2018). Hangover symptoms differ both in frequency of occurrence and in severity, and they may have a differential impact on mood and cognitive or physical performance (Van Schrojenstein Lantman, Mackus, van de Loo, & Verster, 2017).

Over the past decade, several studies have examined the effects of alcohol hangover on cognitive performance and its impact on daily activities. With only a few exceptions (Collins & Chiles, 1980; Streufert et al., 1995; Taylor, Dolhert, Friedman, Mumenthaler, & Yesavage, 1996; Yesavage, Dolhert, & Taylor, 1994), studies consistently showed significant impairment during alcohol hangover on potentially dangerous daily activities such as driving a car (Laurell & Törnros, 1983; Törnros & Laurell, 1991; Verster et al., 2014; Verster, van der Maarel, McKinney, Olivier & de Haan, 2014), riding a bicycle (Hartung et al., 2015), flying an airplane (Morrow, Leirer, & Yesavage, 1990; Morrow et al., 1993; Petros et al., 2003; Yesavage et al., 1994; Yesavage & Leirer, 1986), and job performance such as ship power plant operation Rohsenow, Howland, Minsky, & Arnedt, 2006) or surgery (Kocher, Warwick, Al‐Ghnaniem, & Patel, 2006; Van Dyken, Szlabick, & Sticca, 2013). Nonetheless, a more comprehensive understanding of the alcohol hangover and its impact on cognition is required. Through repetition of traditional tasks used in hangover research, the internal and external validity can be improved, and through the application of novel cognitive tasks, a broader knowledge of the process affected by a hangover can be gained.

Cognition is the ultimate function of the brain (Robbins, 2011) that encompasses how it processes information that play a fundamental role in everyday living (Levitin, 2002). As daily tasks usually incorporate complex skills and abilities, they require adequate functioning of a combination of various aspects of cognitive functioning such as memory, attention, psychomotor skills, and executive functions. In intervention studies, these functions are further influenced by potential mood changes, adverse effects or symptoms accompanying drug administration, or a disease state or condition. Such effects also apply to hangover research, in which both cognitive impairment and mood effects have been demonstrated (Minge, Kollra, & Brieler, 2018). Although it is often unclear whether testing cognitive abilities, self‐reported symptoms, and mood changes in isolation provide an adequate view of performing complex tasks such as driving (Verster & Roth, 2012a, b), these tests do provide detailed insight into which skills and abilities are impaired and may therefore give rise to the use of cognitive modeling in order to ascertain why certain cognitive impairment occurs during an alcohol hangover in the first instance.

As it is important to thoroughly investigate which cognitive functions might be impaired during hangover, and to what extent, the aim of the current study was to investigate memory functioning, several aspects of attention, and mood during alcohol hangover.

## 2 METHODS

### 2.1 Subjects and design

Healthy volunteers were recruited at Ulster University Halls of Residence to participate in a naturalistic study with a between subjects design. Ethical approval for this study was obtained from the ethics committee at Ulster University. Participants were recruited in the morning. As recruitment took place *after* a drinking session (or a night without alcohol consumption) the investigators did not interfere with the participants' alcohol consumption or accompanying behaviors. After signing informed consent, participants who had consumed alcohol the evening before and had an Acute Hangover Scale score above 0 (AHS > 0) were allocated to the hangover group and those who had not consumed alcohol were allocated to the control group. Participants with a breath alcohol reading above zero were excluded from participation. Participants were tested the same morning, shortly after being recruited, between 9 and 11:30 a.m. An incentive for study participation was not offered.

### 2.2 Procedure

Testing took place at the Halls of Residence in order to prevent any traveling to or from the testing location while having an alcohol hangover. Participants completed the AHS (Rohsenow et al., 2007), demographic information was collected, and information was gathered about the number of hours sleep and previous night's alcohol consumption. Thereafter, a cognitive test battery was completed and the Herbert adaption of the Bond and Lader mood scale (Bond & Lader, 1974; Herbert, Johns, & Doré, 1976).

### 2.3 Eriksen's flanker task (Eriksen & Eriksen, 1974)

In this selective spatial attention task, the targets and distracters consist of the letters A and B (15). Distracters are presented at either side of the target and appear either near (1 cm) or far (3.4 cm) from the target. Distracters are either compatible (AAA) or incompatible with the target (BAB). Participants are required to respond to the target letter by pressing an appropriate key as quickly and accurately as possible. Outcome variables include “total errors,” “distance,” and “compatibility” response times. Distance is calculated by subtracting response times (RTs) to far items from RTs to near items, and compatibility is computed by subtracting RTs to compatible items from RTs to incompatible items.

### 2.4 Stroop test (Stroop, 1935)

In this task, words are presented on the screen one at a time in blue, green, red, purple, and brown as used in the original task (Stroop, 1935). Ignoring the text meaning of the words, participants are required to respond to the font color only by using the corresponding buttons on the keyboard provided. Outcome variables include the number of errors and Stroop interference. Stroop interference represents the difference between RTs for congruent (e.g., red presented in red font) and incongruent items (e.g., red presented in green font).

### 2.5 Divided attention test (Tedstone & Coyle, 2004)

In this test (Tedstone & Coyle, 2004), a series of single digits appear in the center of a computer screen at a rate of one per second. When three consecutive odd numbers appeared (e.g., 5, 3, and 7) in the center of the screen, participants are required to respond appropriately using the keyboard in front of them (central, “Z”). Simultaneously, a blue box may appear left, right, below, or above the center of the screen (peripheral). Participants are required to respond when a blue box appears on the screen as quickly and accurately as possible by pressing “M” on the keyboard. Outcome measures included total errors, central RTs, and peripheral RTs.

### 2.6 Free recall

The free recall task consists of 20 words that are presented on the computer screen at a rate of one word every 2 s. In the minute directly following presentation, participants are required to write down as many words as they can remember. The outcome measure is the number of correctly recalled words.

### 2.7 CANTAB—Spatial working memory test

The Cambridge Neuropsychological Test Automated Battery (CANTAB) spatial working memory task requires retention and manipulation of visuospatial information (Cambridge Cognition, 2018a). Participants must touch the colored squares in order to find a blue token. A number of colored boxes are shown on the screen, and the subject should find one yellow “token” in each of a number of boxes and use them to fill up an empty column on the right‐hand side of the screen. Task difficulty varies as the number of boxes can be gradually increased, and the color and position of the boxes changes from trial to trial to prevent predictability. The most efficient strategy is to choose an order to press the boxes and start over in the same order each time a blue token is found. Outcome measures include number of errors (selecting boxes that have already been visited) and strategy. Higher strategy scores indicate poorer use of the best strategy.

### 2.8 CANTAB—Intradimensional–extradimensional set shifting test

This test is a computerized analogue of the Wisconsin card sorting task that features visual discrimination and attentional set formation maintenance, shifting, and flexibility of attention (Cambridge Cognition, 2018b; Gallagher et al., 2011; Kocher et al., 2006; Rohsenow et al., 2006). In this task, participants must use feedback to work out a rule that determines which stimulus is correct. After six correct responses, the stimuli and/or rule changes. Starting with simple stimuli (individually shown white lines and pink shapes) corresponding to intradimensional shifts in rules. Gradually, the task becomes more complex (e.g., white lines overlaid on the pink shapes) also requiring extradimensional rule shifting. Outcome measures are the number of intradimensional and extradimensional errors (i.e., failing to identify the strategy within six trials).

### 2.9 Mood scale

The Herbert, Johns, and Doré mood scale (Herbert et al., 1976), adapted from Bond and Lader (1974) contained the following 18 items that were presented at opposite ends of an 8‐cm line: alert/drowsy, contented/discontented, calm/excited, troubled/tranquil, strong/feeble, mentally slow/quick witted, muzzy/clear headed, tense/relaxed, incompetent/proficient, happy/sad, antagonistic/friendly, interested/bored, withdrawn/sociable, depressed/elated, self‐centered/outward going, well coordinated/clumsy, and lethargic/energetic. Participants were required to place a mark on the line at a position that indicated how they were currently feeling. The raw scores for each line of bipolar items were then derived from the distance of the mark from the item on the left (0–7).

### 2.10 Statistical analysis

Statistical analysis was conducted with SPSS, version 24. Mean (*SD*) were computed for each variable. Participants who consumed alcohol but had a score of 0 at the AHS, that is, indicating the absence of hangover, were excluded from the analysis. Cognitive test results from the hangover group and control group were compared using independent samples *t* tests and considered significant if *p* < .05. For the mood scale, a Bonferroni correction was applied to account for multiple comparisons (differences were considered statistically significant if *p* < .003).

## 3 RESULTS

Ninety‐eight participants were recruited to take part in the study. Fifteen participants withdrew from the study or were absent at the arranged time of testing (i.e., 1 hr after recruitment), and nine participants were excluded because they had a positive breath alcohol test. Seventy‐four university student volunteers (*N* = 39 men and *N* = 35 women) completed the study. Five of these participants had a score of zero on the AHS and were excluded from the analysis. Demographic details of the final sample are summarized in Table 1.

**Table 1:** Demographics and alcohol consumption variables of the hangover group and control group

Demographics	Hangover group	Control group	P value
*N*	35	34	
Male/Female	19/16	18/16	911
Age	22.4 (5.3)	25.1 (6.7)	069
NAge of first drink	15.3 (1.4)	15.8 (2.3)	.240
Hours of sleep	6.3 (2.0)	7.3 (2.1)	.048*
Alcohol consumed on night	13.8 (10.2)	0.0 (0.0)	.000*
Alcohol Hangover Severity	14.2 (11.5)	0.0 (0.0)	.000*
Caffeine (yes/no)	18/17	15/19	.716

* *p* < 0.05.

No significant differences were observed between the hangover group and the control group on gender, age, age of first alcoholic drink, and caffeine consumption. This indicates that the groups were well matched. A mean (*SD*) of 13.8 (10.2) units of alcohol was consumed by participants in the hangover group. Participants in the hangover group slept significantly shorter compared with the control group (*p* = .048), and only about 8.6% of participants went to bed before midnight. In contrast, 45.7% of subjects in the control group reported going to bed at or before 12 a.m. In relation to alcohol consumption, 57.1% of the hangover group and 48.5% of the control group reported drinking on average “once or twice a week.” Consuming alcohol “three to five times a week” was reported by 22.9% of the hangover group and 12.1% of the control group. A percentage of 47.1% of the hangover group and 42.4% of the control group reported consuming alcohol most often in a public house (e.g., local pub). The least popular place to consume alcohol was in a nightclub (hangover group = 20.6%, control group = 12.1%).

Table 2 summarizes the results on the cognitive test battery.

**Table 2:** Summary of the results from the cognitive test battery

Cognitive tests	Hangover group	Control group	P value
Stroop test			
Interference (ms)	401.9 (243.0)	284.82 (231.8)	.040*
Number of errors	5.5 (2.0)	6.3 (4.0)	.290
Eriksen's Flanker test			
Response time (ms)	571.2 (120.9)	505.5 (52.5)	.005*
Number of errors	1.8 (1.5)	2.8 (4.7)	.223
Divided attention test			
Response time (ms)	720.3 (147.0)	689.3 (118.8)	.356
Number of errors	3.1 (3.4)	2.5 (3.4)	.527
CANTAB—Intradimensional–extradimensional set shifting test			
Extradimensional no. of errors	12.5 (10.2)	10.5(10.8)	.434
Intradimensional no. of errors	6.1 (3.4)	8.3 (5.4)	.046*
CANTAB—Spatial working memory test			
Number of errors	22.9 (17.4)	26.0 (20.5)	.515
Strategy score	30.2 (6.1)	32.2 (5.2)	.146
Free recall test			
Total words recalled	7.4 (2.6)	8.7 (2.5)	.038*

* *p* < 0.05.

In the Stroop test, subjects responded significantly faster to congruent words when compared with incongruent words (*p* < .0001). This interference effect was significantly more pronounced in the hangover group compared to the control group (*p* = .040). In the Eriksen flanker task, participants of the hangover group responded significantly slower than participants of the control group (*p* = .005). Significantly fewer words were recalled by the hangover group than the control group (*p* = .038), and more intradimensional errors were made by the hangover group than the control group also (*p* = .046). However, there were no significant differences in divided attention and spatial working memory performance.

Mood assessments are summarized in Table 3. After Bonferroni correction, participants in the hangover state reported significantly higher levels of drowsiness and clumsiness. Other mood scores did not significantly differ between the groups.

**Table 3:** Subjective mood ratings

Mood ratings	Hangover group	Control group	P value
Alert/drowsy	3.0 (1.9)	1.5 (1.4)	.000*
Contented/discontented	1.4 (1.5)	1.4 (1.7)	.966
Calm/excited	1.4 (1.2)	1.2 (1.3)	.458
Troubled/tranquil	4.1 (1.7)	4.0 (1.9)	.844
Strong/feeble	2.7 (1.7)	1.8 (1.5)	.033
Mentally slow/quick witted	3.0 (1.8)	3.7 (1.8)	.103
Muzzy/clear headed	3.1 (2.0)	4.1 (2.0)	.037
Tense/relaxed	4.02 (1.8)	4.2 (2.1)	.940
Attentive/dreamy	2.9 (1.9)	2.2 (1.9)	.158
Incompetent/proficient	4.1 (1.6)	4.1 (1.8)	.894
Happy/sad	1.2 (1.4)	1.4 (1.8)	.761
Antagonistic/friendly	5.2 (1.2)	5.0 (1.7)	.697
Interested/bored	1.3 (1.5)	1.4 (1.8)	.787
Withdrawn/sociable	4.1 (1.9)	4.4 (1.7)	.450
Depressed/elated	3.8 (1.4)	4.4 (1.7)	.167
Self‐centered/outward‐going	3.5 (1.6)	3.9 (1.7)	.347
Well‐coordinated/clumsy	3.6 (1.8)	2.0 (1.9)	.000*
Lethargic/energy	2.6 (1.7)	3.8 (1.8)	.008

* *p* < 0.003, after Bonferroni correction.

## 4 DISCUSSION

Our results indicate that response times in both spatial (Eriksen's flanker task) and dimensional (Stroop) selective attention tasks are impaired during alcohol hangover. Significantly higher levels of drowsiness and clumsiness were reported that are illustrative of performance affecting increased levels of fatigue that are commonly experienced during the hangover state (Penning et al., 2012).

Several studies have examined attention and memory functioning during hangover. Most of these studies reported the absence of significant hangover effects on divided attention (Chait & Perry, 1994; Collins & Chiles, 1980; Finnigan, Hammersley, & Cooper, 1998; Lemon, Chesher, Fox, Greeley, & Nabke, 1993; Seppälä, Leino, Linnoila, Huttunen, & YIikahri, 1976). However, the duration of most divided attention tests, including the one used in the current study, is limited to a couple of minutes. Studies with divided attention test of longer duration (e.g., 15 min or more) did show significantly more tracking errors in the hangover state (Roehrs, Yoon, & Roth, 1991). A previous study from our institute using the same divided attention task (McKinney, Coyle, & Verster, 2012) did find significantly slower response times in the hangover condition. A possible explanation for this discrepancy in results may be the fact that the study by McKinney et al. (2012) applied a within subjects design as opposed to the between group design of the current study. In line with the current study, McKinney et al. (2012) also observed significantly slower response times on the Stroop task and Eriksen's flanker test. They found no significant differences between hangover and control condition on a spatial attention test (which was not included in the current study). In several other studies, no significant hangover effects were found on tests of vigilance performance and/or sustained attention as assessed with the continuous performance test (Rohsenow et al., 2010; Howland et al., 2010) the psychomotor vigilance task (Rohsenow et al., 2010), or the Mackworth clock test (Lemon et al., 1993; Verster, Van Duin, Volkerts, Schreuder, & Verbaten, 2003). However, Howland et al. (2010) did report significant impairment on the psychomotor vigilance task during hangover.

This literature summary illustrates that the extent to which attention is affected by alcohol hangover depends on the type of attention that is investigated. It also shows that there is discrepancy between the outcomes of studies that used the same tests. These differences are most likely due to methodological differences with regards to design (e.g., using a between subjects or with subjects design), differences in sample size and power, and to what extend drinking behavior and other activities were controlled (naturalistic designs vs. controlled laboratory studies). Nevertheless, in more complex attention tasks, the effects are of greater magnitude and more likely to reach statistical significance. Some of the applied divided attention tests in previous research were of short duration and required relatively little cognitive capacity to be performed. In these instances, motivation and increased effort by the participant may have been able to counteract the impairing attention effects that accompany the hangover state.

With regard to memory functioning, the current study only tested immediate free word recall an observed a significant difference between the hangover and control group. In contrast, most previous studies have not found significant hangover effects on short term or working memory tasks (Chait & Perry, 1994; Finnigan et al., 1998; McKinney et al., 2012; Verster et al., 2003). However, McKinney and Coyle (2004) did find significant impairment in immediate free word recall. Of note, significant impairments in delayed free word recall have been reported to be present during the alcohol hangover Verster et al., 2003). Delayed recognition was significantly impaired in two studies McKinney & Coyle, 2004; McKinney et al., 2012) but not in another study (Verster et al., 2003). Unfortunately, delayed recall and delayed recognition were not assessed in the current study.

Strengths of the study include its relatively large sample size compared with many other hangover studies and the fact that validated standardized cognitive tests were used to assess attention and memory functioning. Another strength was the use of a naturalistic design in which participants were recruited *after* they had a night of alcohol consumption or an alcohol‐free night. This methodology ensured that participants have acted 100% naturally without any involvement of investigators or the knowledge that they were participating in an experimental study. The latter is an important advantage if one aims to mimic a routine natural drinking occasion instead of a controlled and observed drinking session in the laboratory. Activities, mood and drinking behavior were thus not influenced by the researchers.

In terms of methodological shortcomings, the lack of control can be regarded as a limitation of this naturalistic study, as it introduces unwanted variability between participants. For the same reason, the between subjects design can also be regarded a limitation. Variability between participants may have impacted the outcome of the statistical analysis to a greater extent than would have been observed when applying a within subjects design. Although demographic characteristics of the hangover group and control group matched well in the current study, it is possible that the groups may have varied at baseline in relation to cognitive skills and abilities. It would therefore be good to replicate the current study using a cross over design in which the same participants are tested both on a hangover day and an alcohol‐free control day. Finally, *N* = 9 participants with a positive BAC reading were excluded from participation, which was considered good practice at the time the study was conducted. However, current consensus (Van Schrojenstein Lantman et al., 2016) defines that the alcohol hangover is “…starting when blood alcohol concentration (BAC) approaches zero.” With this knowledge, future studies should also include participants with a positive BAC reading in the study and statistical analysis.

Taken together, this study contributes to our understanding of the impact of an alcohol hangover on memory and attention performance. With consideration of the methodological advantages and disadvantages of the current study, it is concluded that selective attention is significantly impaired during alcohol hangover. Other forms of attention, immediate free recall and spatial working memory did not differ significantly between the hangover and control groups. Future studies should aim to replicate and extend the current findings, preferably using a within subjects design.

## AUTHORS' CONTRIBUTIONS

L. D., K. C., and J. V. made substantial contributions to conception and design; L. D. analyzed the data; L. D. and J. V. drafted the manuscript; and all authors revised it critically for important intellectual content and approved the final version of the manuscript.

## AVAILABILITY OF DATA AND MATERIALS

The dataset analyzed during the current study is available from the corresponding author on reasonable request.

## CONFLICT OF INTEREST

Over the past 3 years, Joris Verster has received grants/research support from the Dutch Ministry of Infrastructure and the Environment, Janssen, Nutricia, and Sequential and acted as a consultant/advisor for Clinilabs, Janssen, Jazz, More Labs, Red Bull, Sen‐Jam Pharmaceutical, Toast!, Vital Beverages, and ZBiotics. The other authors have no potential conflict of interest to disclose.
